# New Appliances and Things Medical

**Published:** 1907-06-22

**Authors:** 


### NEW APPLIANCES AND THINGS MEDICAL.

[We shall be glad to receive at our Office, 28 & 29 Southampton Street, Strand, London, W.C., from the manufacturers, specimens of all new preparations and appliances which may be brought out from time to time.]

### ANALGESIC BALSAM.

(Dr. Bengué and Co., London Office, 91 Great Titchfield Street, London, W.)

This is a mixture of menthol, synthetic methyl salicylate and pure lanoline. It is neutral in reaction, and possesses decided antiseptic properties, and has been recommended as an ointment in cases of neuralgia. The anæsthetic value of menthol is well recognised, and this combination with methyl salicylate, which in itself has some analgesic power, is remarkably efficacious in cases where such an ointment is required. Full directions are enclosed with the patent collapsible tubes in which the balsam is put up. A word of caution should be added as to the irritating effect which menthol is likely to produce on parts which are highly inflamed.

### "SOLUROL."

(Allen and Hanburys, Ltd., 37 Lombard Street, London, E.C.)

Thyminic acid has been recommended in the treatment of gout, owing to the fact that a solution of the acid is a solvent for uric acid. It has been found useful in severe cases of gout, and in rheumatic conditions, and it is likely to prove of service in all cases where there is any evidence of uratæmia or uratosis. "Solurol" is the trade name under which Messrs. Allen and Hanbury supply the drug. It is put up in four-grain tablets, uncoated. The tablets are pale fawn grey in colour, easily soluble in the mouth, and not unpleasant to the taste, their flavour reminding one of a weak meat-extract. The dose is one to two tablets (4-8 grains) three times a day after food.

